# Compact Origins and Where to Find Them: ORC's Guide to Genome‐Wide Licensing

**DOI:** 10.1002/bies.70018

**Published:** 2025-05-19

**Authors:** Christian Speck, Luitpold Maximilian Reuter

**Affiliations:** ^1^ Institute of Clinical Sciences Faculty of Medicine Imperial College London London UK; ^2^ MRC London Institute of Medical Sciences (LMS) London UK; ^3^ Institute of Molecular Biology (IMB) gGmbH Mainz Germany

**Keywords:** budding yeast, ChIP‐Exo, DNA licensing, DNA replication, MCM2‐7, ORC, origins

## Abstract

Origin licensing is the first step in the fundamental process of DNA replication, which ensures the accurate transmission of an organism's genetic information. Studies in budding yeast have provided crucial insights into replication origins, revealing sequence‐specific features and structural DNA elements guiding helicase loading. Here, we review the recent advances in our understanding of DNA replication origin licensing, focusing on insights into origin architecture and advancements in high‐resolution sequencing. Progress in the field demonstrates that origins are compact units that load an individual MCM2‐7 double hexamer, which in turn causes steric occlusion of the origin recognition complex (ORC) binding site. We discuss why, in addition to the DNA sequence, DNA shape, DNA flexibility, and correct spacing of A‐ and B2‐elements are crucial for efficient helicase loading. These recent findings provide a mechanistic explanation for the regulation of genome‐wide origin licensing and reveal fundamental principles of MCM2‐7 helicase loading.

## Graphical abstract

Origin licensing is a key requirement for any eukaryotic cell to initiate DNA replication in S‐phase. Here, we review the most recent findings in the orchestrated action of the origin recognition complex (ORC) to sequentially load two MCM2‐7 helicases onto DNA with a focus on origin architecture and sequencing‐based methods.

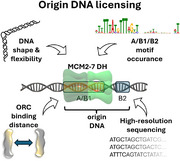

## 1 Introduction

The duplication of DNA must be of the highest quality to preserve genetic information. Mistakes during this process lead to the accumulation of mutations, ultimately promoting disease and aging. DNA replication is a two‐step process, starting with the loading of the replicative helicase at a single (bacteria) [1], hundreds (yeast) [2], or thousands (human) [3] of replication origins followed by activation of the helicase. In eukaryotes, this process is regulated by cyclin‐dependent kinases (CDKs) [4]. G1‐phase specific kinases are permissive for helicase loading, while S‐phase specific kinases promote helicase activation, halting helicase loading at the same time [5, 6]. This switch guarantees that each piece of DNA is only replicated once [7]. Misregulation of this process results in replication stress and genome instability, promoting cancer and ageing [8].

Over the last 40 years, the genetic, genomic, biochemical, and structural analysis of bacterial, yeast, and human DNA replication has generated fundamental insights. The bacterial DNA replication systems are simpler, as most bacteria contain only a single replication origin on their individual circular chromosome [1, 9]. Here, replication fork assembly is controlled by the initiator DnaA, an ATP‐binding protein [10]. Increasing concentrations of ATP‐DnaA allow the formation of an extended complex across the replication origin [11, 12, 13]. This is directed by sequence‐specific DNA binding sites, termed DnaA boxes [14]. Once sufficient levels of ATP‐DnaA have accumulated, the complex unwinds an AT‐rich region, which allows for the loading of two DnaB helicase hexamers and the formation of replication forks to initiate DNA synthesis [1, 10]. To limit rereplication, bacteria have evolved several mechanisms to limit DnaA‐dependent DNA unwinding of the replication origin, including the rapid transformation of ATP‐DnaA into inactive ADP‐DnaA [15, 16, 17, 18].

**FIGURE 1:**
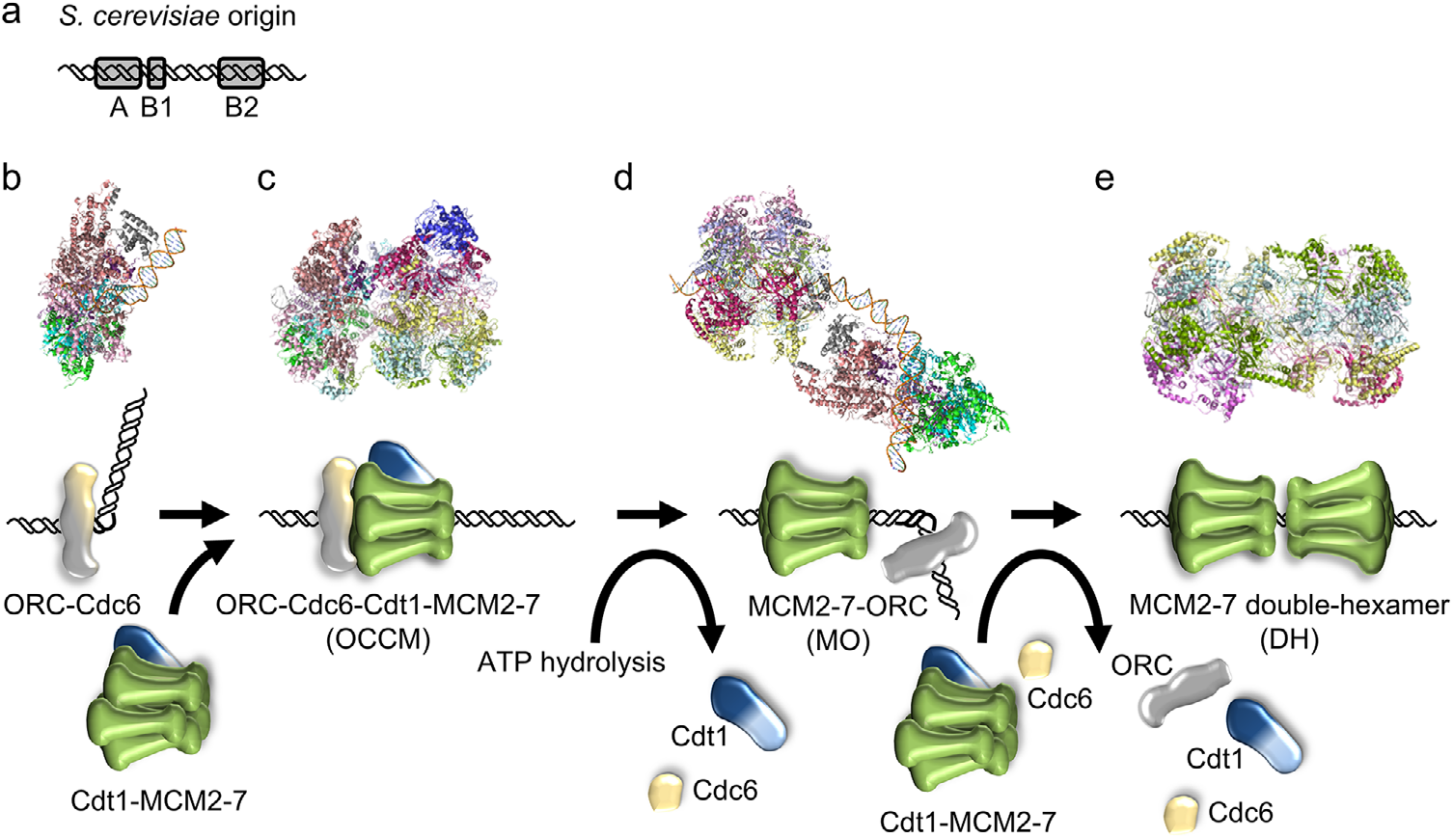
Budding yeast DNA licensing. (a) Simplified *S. cerevisiae* origin architecture. Origins typically consist of an A‐, B1‐, and B2‐element to load a stable MCM2‐7 double hexamer onto dsDNA. (b) ORC and Cdc6 form a complex on DNA and bend the DNA (PDB 7MCA [31]). (c) Cdt1‐MCM2‐7 becomes recruited, DNA is inserted, and the MCM2‐7 ring partially closes around DNA, resulting in the OCCM complex (PDB 5V8F [76]). Mcm4 ATP‐hydrolysis results in complete MCM2‐7 ring closure and Cdt1 and Cdc6 release. (d) ORC becomes repositioned to the other (N‐terminal) side of the MCM2‐7 hexamer, generating the MCM2‐7‐ORC complex (MO, PDB 6RQC [46]). (e) Following the recruitment of Cdc6, Cdt1‐MCM2‐7, and ATP‐hydrolysis‐dependent release of the helicase loading factors, the MCM2‐7 double‐hexamer forms around DNA (DH, PDB 5BK4 [50]).

Budding yeast has been the premier model system for studying eukaryotic DNA replication, generating outstanding insights into the process [5, 19, 20]. Here, specific DNA sequences serve as replication origins—termed autonomously replicating sequences [21, 22, 23]. They encode genetically defined elements, termed A‐, B1‐, and B2‐elements (Figure 1a). The A‐element is essential and the B‐elements are important for the origin function [24]. Biochemical analysis revealed that the A‐ and B1‐elements serve as binding sites for the origin recognition complex (ORC) [25]. In budding yeast, ORC is a six‐subunit complex (Orc1‐6), which binds and hydrolyses ATP [25, 26, 27]. In the late G2/M‐ and early G1‐phase, ORC recruits Cdc6 to the origin, which results in an extended DNA footprint [28, 29, 30]. ORC‐Cdc6 encircles and bends DNA on one side of the complex [31, 32, 33] (Figure 1b). This DNA bending allows the recruitment of the minichromosome maintenance (MCM2‐7) complex and Cdt1 [34]. MCM2‐7 represents the heterohexameric core of the replicative helicase in all eukaryotes [35, 36]. In budding yeast, Cdt1 and MCM2‐7 form a stable complex [37]. MCM2‐7 proteins have C‐terminal extensions, which establish connections with ORC‐Cdc6. The Mcm3 C‐terminus initiates complex assembly by binding to Orc1 and Cdc6 [34, 38, 39], while the Mcm6 C‐terminus can only make contact with Orc4 and Orc5 when Cdt1 is present [40, 41]. Helicase loading involves the insertion of the spiral‐shaped MCM2‐7 hexamer via an Mcm2/Mcm5 interface opening onto DNA (Figure 1c) [34, 42–44]. This leads to ring closure around DNA and the induction of Mcm4 ATP‐hydrolysis, which alters the MCM2‐7 structure [45, 46]. In turn, Cdc6 and Cdt1 are released, and ORC becomes repositioned to the MCM2‐7 N‐terminus (Figure 1d) [46, 47, 48, 49]. Consequently, ORC recruits another Cdc6 and Cdt1 to promote the recruitment of a second MCM2‐7 hexamer [46, 47]. The final product is a salt‐stable MCM2‐7 double‐hexamer (DH), which encircles double‐stranded (ds) DNA (Figure 1e) [5, 19, 20, 50, 51]. This complex is initially inactive until it becomes activated by the Dbf4‐dependent kinase Cdc7 (DDK) [52, 53, 54, 55, 56]. DDK targets the Mcm2, Mcm4, and Mcm6 N‐termini for phosphorylation, which also alleviates an inhibitory activity in Mcm4 [57]. Consequently, Sld3‐Sld7 is recruited, promoting Cdc45 binding [6, 58, 59]. CDK phosphorylation of Sld3 and Sld2 promotes the recruitment of GINS (acronym for: go‐ichi‐ni‐san; Sld5, Psf1, Psf2, and Psf3), polymerase epsilon, Dpb11, and Sld2 [60, 61, 62]. This results in the formation of a stable Cdc45‐MCM2‐7‐GINS (CMG) complex, which represents the core of the eukaryotic replication fork [5, 63, 64]. Consequently, Mcm10 activates the CMG, which triggers the assembly of the full replication fork and DNA synthesis [65, 66, 67, 68].

Human DNA replication is currently less understood, in part, as it has not yet been fully reconstituted. Genomic analyses have revealed that human DNA replication origins do not contain an overall recognition sequence but a multitude of features, including epigenetic marks, specific proteins, DNA structures, and DNA motifs [69, 70, 71, 72, 73]. Indeed, human Orc4 is missing a short helical insertion in its DNA binding motif that allows yeast ORC to bind to specific ARS sequences [74, 75, 76]. Instead, human and metazoan ORC generally bind DNA in a largely sequence‐independent manner [77, 78, 79, 80, 81]. Work in yeast has identified that DNA structure could be involved in the selection of replication origins [32, 82]. To determine if this is also the case in the context of human origins requires further work. The recent reconstitution of the first step of human DNA replication, MCM2‐7 double‐hexamer formation, revealed that human ORC6 does not form a stable complex with ORC1‐5 and is not essential for replicative helicase loading [79, 80]. Instead, it is associated with the pre‐RC only after ATP‐hydrolysis and increases the efficiency of the reaction [78, 79, 80]. The end product of human DNA licensing is also the MCM2‐7 double‐hexamer on dsDNA [78, 79, 80]. In contrast to yeast, the human complex displays limited DNA unwinding in its central section [79, 83]. The steps that lead to the assembly of the human CMG are still being researched, but human CMG has been purified and used for the reconstitution of the replication fork [84, 85].

The following sections focus on budding yeast DNA replication, where we review the recent advances in next‐generation sequencing, which have deepened our understanding of DNA replication origin licensing in vivo. We emphasise the insights into origin architecture and the origin‐bound protein complexes involved.

### 1.1 Budding Yeast Origin Architecture

**FIGURE 2:**
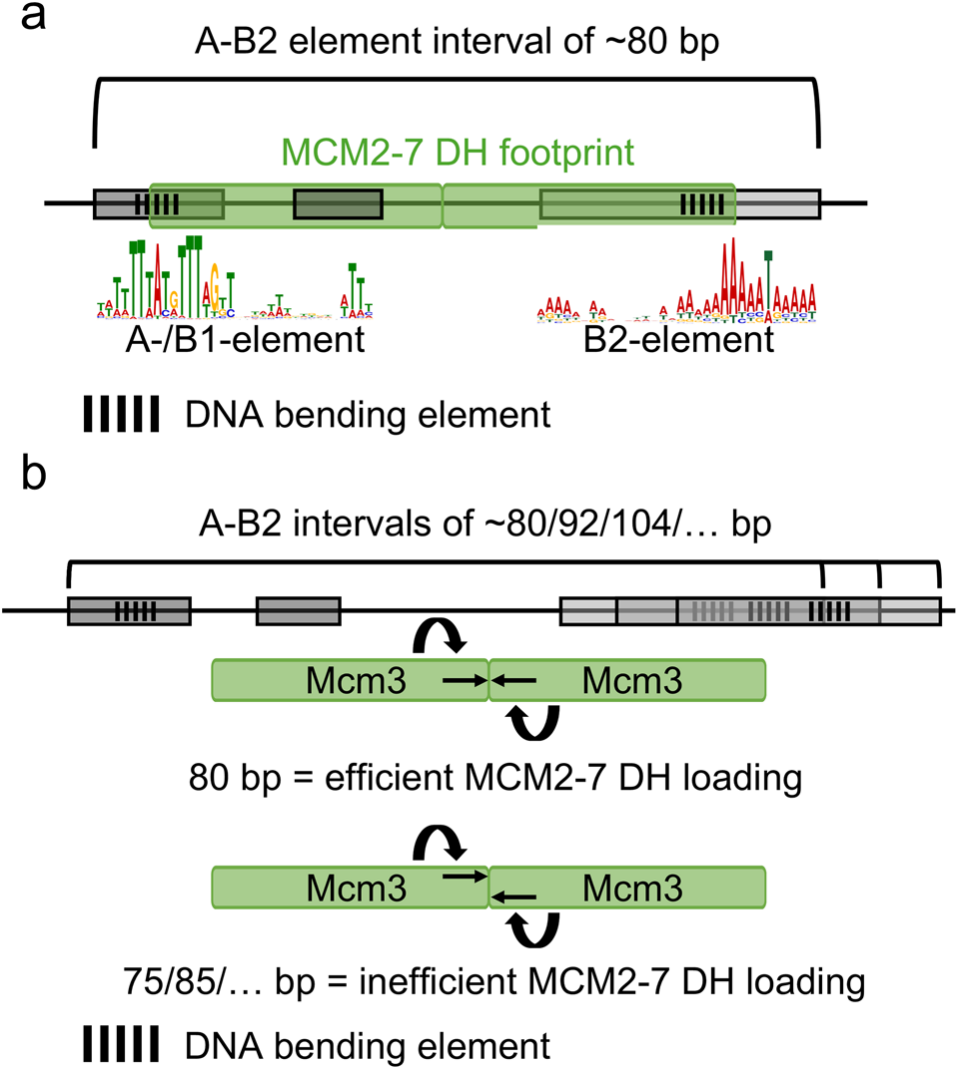
Simplified budding yeast origin architecture. (a) Budding yeast replication origins contain conserved A‐, B1‐, and B2‐elements. The sequence motif of the A‐/B1‐element and the B2‐element are shown. The DNA sequences encode for specific DNA shapes, which support ORC‐dependent DNA bending. Frequently, the A‐/B1‐ and B2‐elements together take up 80 bp. (b) The A‐/B1‐ and B2‐elements can be found in varying distances, enriched for ∼12/13 bp intervals. An optimal distance is associated with optimal MCM2‐7 loading. We suggest that the ideal distance ensures the correct alignment of the two MCM2‐7 hexamers, where the Mcm3 N‐termini of each hexamer faces each other.

Budding yeast replication origins contain, in general, AT‐rich sequences with conserved motifs at their heart [86]. The specific sequences are only conserved within the *Saccharomyces* clade, with no ORC recognition sequence identified in any other eukaryotic model organism [87]. The sequence specificity of budding yeast allowed the field to discover many fundamental principles in DNA replication. Most origins contain A‐, B1‐, and B2‐sequences, while some also contain a B3‐sequence [24, 86]. While the A‐ and B1‐sequences serve as binding sites for ORC and Cdc6 [25, 28], the function of the B2‐element was not clear for the longest time. Finally, the B3‐element is the binding site for the transcription factor Abf1 [24, 88]. It is involved in chromatin reorganisation, transcriptional control, and DNA replication [24, 89, 90]. Initially, the B2‐element has been proposed to function as a DNA unwinding element (DUE) for the MCM2‐7 helicase or as a second ORC binding site [91, 92]. However, understanding the B2‐element function was hampered by its poor sequence conservation among replication origins [93]. Also, while the A‐ and B1‐elements can be observed at a fixed distance, the B2‐element can be found at various distances from the A‐element [93, 94]. Thus, it was more challenging to identify and study. The recent high‐resolution genomic analysis of the MCM2‐7 DH provided new insights into the B2‐element [82]. It was discovered that the B2‐element can be found adjacent to the MCM2‐7 DH, on the opposite DNA strand of the A‐element (Figure 2a). This allowed deeper sequence analysis of the B2 motif, which revealed a sequence similar to ORC binding sites, except for conserved GC bases [82]. Thus, the data suggest that the B2‐element functions as the secondary ORC binding site for loading the second MCM2‐7 hexamer. Crucially, this is consistent with work by the Diffley lab, who engineered an origin with two inverted A‐/B1‐elements to establish quasi‐symmetrical MCM2‐7 loading in vitro and showed origin function in vivo [94]. While the Diffley lab found that a 70 bp spacer between the two A‐/B1‐elements worked optimally [94], we found that the distance between the B2‐element and MCM2‐7 DH was associated with a repeating ∼12/13 bp interval pattern and correlated with MCM2‐7 loading efficiency, suggesting that specific distances are favored for helicase loading [82] (Figure 2b). Therefore, we suggest that the optimal distance could promote the correct rotational arrangement of the MCM2‐7 hexamers, which allows the establishment of the DH interface, where both hexamers are aligned via the Mcm3 N‐termini (Figure 2b) [82].

In the ORC‐DNA structure, the DNA is bent dramatically, leading to local deformation of DNA, such as widening and narrowing of the minor groove width (Figure 1b) [31, 32, 95]. Indeed, the propensity to form a wider minor groove can be observed in the DNA sequence of the A‐ and B2‐elements, indicating that the DNA itself has an altered shape [32, 77, 82]. Concomitantly, other measures of deformation, such as helical twist, propeller twist, roll, or electrostatic potential of the minor groove, provided further evidence of major distortion of the origin DNA, even in the absence of ORC. Interestingly, in the case of the B2‐element, the structural change was associated with efficient MCM2‐7 loading onto origins, suggesting that DNA bending is functionally important and could be a criterion to regulate helicase loading efficiency [82].

In summary, budding yeast replication origins are recognised based on specific DNA sequences and structures. The sequence‐specific aspect makes yeast unique within eukaryotes, while higher eukaryotes frequently use additional features to target origins [69].

### 1.2 To Load or Not to Load: ORC's Licensing Dilemma

Theoretically, a single MCM2‐7 double hexamer loaded onto each origin of replication should be sufficient to complete the replication of the genome. However, it was shown in many organisms that MCM2‐7 protein levels exceed ORC molecules and the number of origins in the cell [96, 97, 98, 99]. This is historically known as the “MCM paradox” and directly raises questions regarding the function and loading mechanisms of MCM2‐7 into DHs. In principle, the abundance of MCM2‐7 molecules opens two pathways for licensing in vivo. On the one hand, “processive” licensing would enable multiple MCM2‐7 complexes to be loaded by a single ORC subsequently across origins, with some comprised of multiple adjacent MCM2‐7 DHs and other origins containing only a single or no DH. Repetitive loading has indeed been observed in vitro using purified proteins and plasmid DNA [47, 100]. in vivo, evidence for multiple loaded MCM2‐7 DHs came from MCM2‐7 chromatin immunoprecipitation and sequencing (ChIP‐Seq) studies [101].

On the other hand, “distributive” helicase loading would allow a maximum of one DH per origin. Evidence for this mode of licensing comes from chromatinised in vitro licensing assays where MCM2‐7 DH movement was inhibited [102] and in vivo and in vitro footprinting experiments [28, 29, 30]. Similarly, in vivo MCM2‐7 high‐resolution binding analyses have identified single MCM2‐7 DHs at replication origins [82, 103, 104]. In a recent genome‐wide study, we identified the molecular footprints of ORC and MCM2‐7 in budding yeast and found that they overlap. Within a cell population, MCM2‐7 loading is most efficient at a subset of origins. Concomitantly, ORC binds these origins most efficiently in G2 phase and becomes largely replaced with MCM2‐7 in G1‐phase [82]. In addition, we observed that ORC was distributed to less efficient origins once licensing was underway. This suggests that upon ORC‐dependent loading of the MCM2‐7 DH, ORC becomes released and cannot rebind to the origin, as MCM2‐7 occludes its binding site. Indeed, when MCM2‐7 loading was blocked, either through mutation of Cdc6 (no activation of licensing) or Mcm5 (defective helicase complex), ORC was retained at the origins in G1‐phase. In summary, this suggests that ORC is displaced from origins during MCM2‐7 helicase loading, promoting distributive genome‐wide licensing, consistent with previous reports [103, 105].

Lastly, we found that single and static MCM2‐7 DH are loaded onto ∼400 origins in budding yeast [82]. Interestingly, while single footprints for ORC and MCM2‐7 DHs were found, the accumulated signal for ORC and MCM2‐7 was not the same across all identified origins, pinpointing cell‐to‐cell variations in binding and licensing efficiency within a population of cells, particularly for dormant and less efficient origins. However, it has been observed that MCM2‐7 DHs can slide away from the origin DNA in vitro, thereby allowing multiple rounds of DH loading [20]. In addition, using an engineered origin in vivo, it was shown that RNA polymerase II can push MCM2‐7 DHs away from the origin DNA, freeing it for another round of licensing [106, 107]. Furthermore, when multiple ORC binding sites are in close proximity, several MCM2‐7 DHs can be found locally, with each ORC loading one DH [82]. Taken together, the loading of MCM2‐7 complexes onto origins is limited to a single DH occupation per origin. Complete licensing of the genome results in excess of MCM2‐7 DHs on DNA, as many origins will remain dormant in an unperturbed S‐phase. Nonetheless, a surplus of licensed origins represents a safeguard mechanism to guarantee total and faithful replication of the genome.

The distributive licensing model is attractive for several reasons: First, once an MCM2‐7 DH is loaded, ORC is released from its binding motif to scan the DNA for other origin sequences with equal or lower sequence conservation [102]. The overlap between ORC and MCM2‐7 binding sites exemplifies a tightly regulated system that balances precision with efficiency in DNA licensing. Even more, the overlapping binding sites, together with the displacement of ORC by MCM2‐7, reveal that origin licensing is a self‐limiting process aiming for distributive DNA licensing. Thus, limited amounts of ORC in the cell can guarantee complete licensing of the genome. Furthermore, the dynamic redistribution of ORC allows it to bind to non‐origin sites where it could fulfill additional functions in chromatin organisation or transcription regulation [108, 109].

Second, single DH loading is plausible in the chromatinised environment of the genome. The yeast genome is occupied by nucleosomes in a dense packing order, with an average center‐to‐center distance of ∼165 bp, leaving around 15–30 bp of linker DNA between neighboring nucleosomes [110]. Thus, this argues that space for origins is limited. We found that the average origin with its motifs makes up ∼80 bp in length and that the MCM2‐7 DH covers ∼64 bp—highlighting that MCM2‐7 loading sites are compact [82]. However, helicase activation factors would require additional space to activate replication forks during the onset of the S‐phase. Together, this showcases the compact architecture of the conserved origin elements and renders processive licensing a difficult task. Multiple DHs could only fit onto a single origin if nucleosomes were disassembled or moved, for example, through nucleosome remodelers, as the helicase motor is inactive until helicase activation in S‐phase. Yet, it was shown that MCM2‐7 DHs interact with nucleosomes at origins and that origins have well‐defined flanking nucleosomes, allowing limited movement during licensing [104, 111].

Third, if multiple DHs were present on a single origin, one or more might be activated. This could either lead to the activation of a single DH that pushes the remaining dormant DHs ahead of the replication fork, or cause the establishment of multiple, local replication forks. These would require immediate termination as soon as the oncoming CMGs meet to prevent over‐replication. Activation and termination within a single origin would be futile, have a high energy expenditure, and would locally trap limited activation factors to high‐efficiency origins driving replication. Instead, if a single DH is already sufficient to license an origin, this DH will be used for activation and replication fork establishment.

The aforementioned points can also explain parts of the long‐standing MCM paradox and the energetic expenditure of producing and loading surplus MCM2‐7 complexes onto DNA as a safeguard for genome stability. Sub‐stoichiometric ORC allows the loading of excess, soluble MCM2‐7, forming single DHs at many origins while keeping energy costs at a minimum. Finally, distributive licensing of origins would also be beneficial for higher eukaryotes to prevent over‐licensing of highly efficient origins while promoting balanced and even licensing throughout the genome. Just recently, it was found that the chromatin life cycles, that is, loading and unloading of MCM2‐7 and its loader ORC are similar in both budding yeast and human cells, inviting speculations regarding the mechanisms of origin licensing for this evolutionary conserved machinery [112].

Taken together, the distributive licensing model can explain mechanistically how origin licensing is realised across the genome without the need for additional regulation, as it employs a self‐limiting process that is hardwired into the budding yeast DNA.

### 1.3 Approaches to Interrogate Genome Licensing

The questions of how origins are defined, licensed, and activated have been at the center of replication research for more than three decades. Naturally, in vitro reconstitution and in vivo analyses have, in parallel, provided valuable insight into the detailed mechanisms underlying origin licensing in various organisms [19, 20, 24, 48, 51, 93, 104]. While reconstitution assays and in vitro footprinting mostly use naked plasmid DNA, in vivo analyses frequently rely on chromatin fractionation, immunoprecipitation, and high‐throughput sequencing techniques to characterise licensing‐relevant complexes and DNA binding sites [19, 21, 101, 113, 114]. In particular, ChIP‐based techniques suffered from limited resolution for a long time due to several complications. These include DNA‐protein crosslinking efficiency, epitope masking, antibody availability and specificity, non‐specific interactions, or DNA shearing efficiency [115, 116]. That said, the number of reported origins varies from ∼200–1600 origins in budding yeast, with most reports agreeing on ∼400 confirmed origins, while the remaining origins are used less frequently or become activated in the context of DNA damage [2, 82, 103, 104, 111, 114, 117].

**FIGURE 3:**
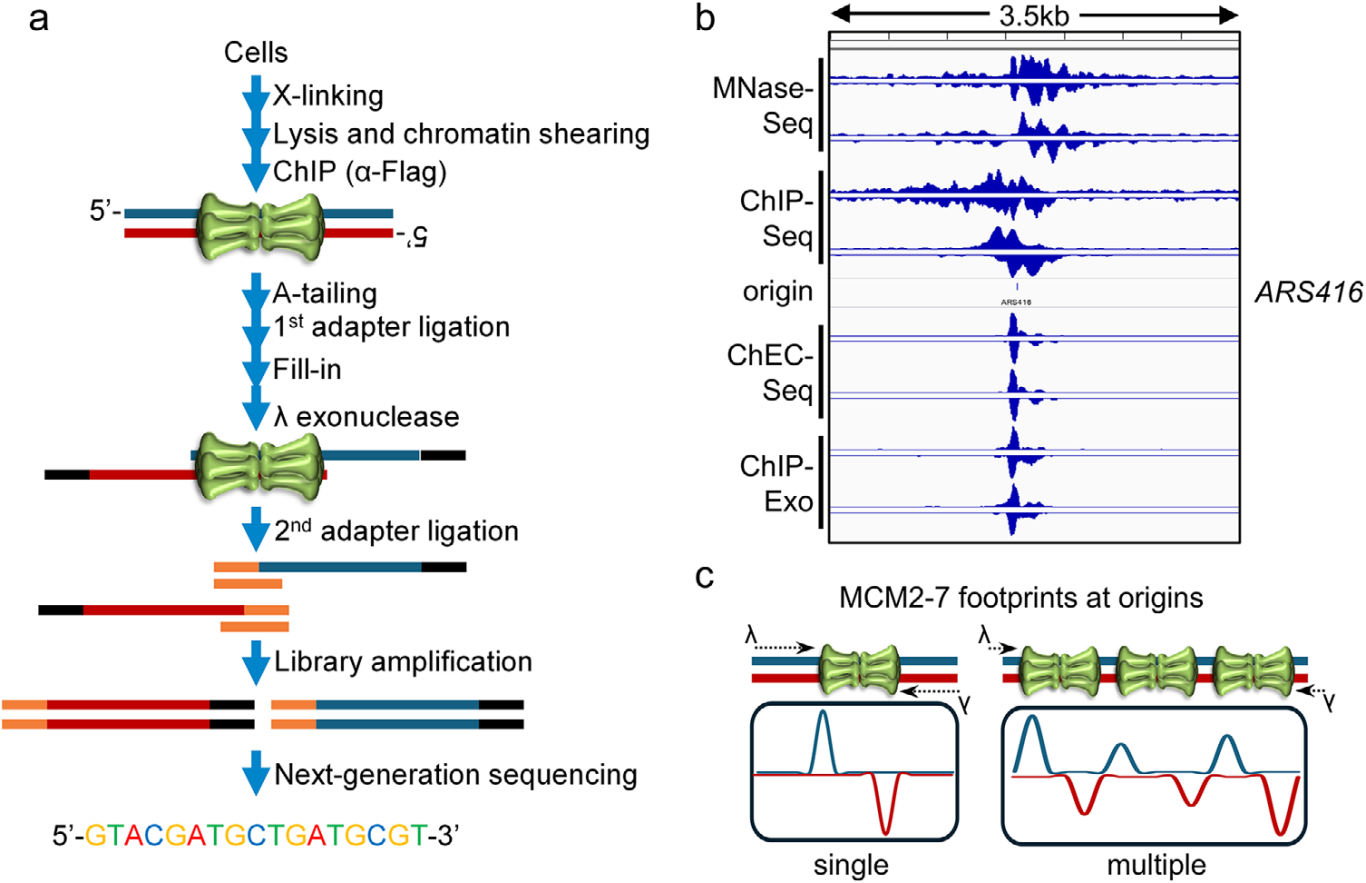
ChIP‐Exo in DNA replication research. (a) Schematic workflow of ChIP‐Exo sample preparation for next‐generation sequencing. Steps include formaldehyde cross‐linking, immunoprecipitation, on‐bead first adapter ligation, and digestion with exonuclease. This is followed by clean‐up, second adapter ligation (splint ligation), and library amplification. (b) Raw data comparison of various MCM2‐7 mapping techniques. MNase‐Seq [119], ChIP‐Seq [104], ChEC‐Seq [103], and ChIP‐Exo [82] traces are visualised in IGV (ver. 2.14.1, https://igv.org/) for *ARS416* within a window of 3.5 kb). (c) ChIP‐Exo tag analysis (footprints) can distinguish between single and multiple, adjacent complexes. The strongest signal is found at the first protein‐DNA contact. Lambda exonuclease access is highlighted by black arrows.

Having said that, one of the recent additions to the pool of sequencing‐based techniques is ChIP‐Exo, a ChIP‐derived technique that includes on‐bead digestion of cross‐linked, precipitated, and adapter‐ligated DNA with Lambda exonuclease (Figure 3a) [113]. Then, DNA is eluted from beads, crosslinks are reversed, and purified fragments barcoded before sequencing. The digestion of DNA (5′‐3′ direction only) to the edge of the protein‐DNA interaction site improves sensitivity and increases positional resolution to a near base‐pair level since most signal accumulates at specific DNA sites with very similar 5′‐ends (Figure 3a,b) [113, 118]. When comparing to published MNase‐Seq [119], ChIP‐Seq [104], or ChEC‐Seq (chromatin endogenous cleavage) [103] datasets of MCM2‐7, ChIP‐Exo [82] produced very sharp, localised peaks similar to the latest MNase‐based MCM2‐7 mapping technique, ChEC‐Seq. Footprints of around 64 bp can already be identified in the aligned raw tracks directly located over the A‐element of an efficient origin (*ARS416* or *ARS1*, Figure 3b). Only these most recent high‐resolution sequencing techniques unified in vitro and in vivo footprints for ORC and MCM2‐7 for the first time [82, 103]. Yet, ChIP‐Exo has a unique advantage over techniques using un‐crosslinked samples or MNase cleavage (ChEC‐Seq [103] or CUT&RUN [120]) as it can distinguish DNA footprints from single and multiple adjacent protein complexes, for example, MCM2‐7 DHs, due to its exonuclease digestion step (Figure 3c). The low background of this technique, due to the combination of sonication, on‐bead adapter ligation, and exonuclease patterning, further allows the evaluation of multiple footprints in a population, that is, through the detection of several adjacent footprints (single or multiple local binding events) or footprints of various sizes (different binding modes, Figure 3a,c). The use of protein tags (5xFLAG, GFP, etc.) over antibodies raised against individual proteins has also increased specificity and robustness in the detection of replication‐relevant complexes in various assays [82, 121].

Over the last few years, more ChIP‐Exo dedicated software and algorithms have been published, simplifying downstream analyses as classical ChIP‐Seq analysis pipelines are unsuitable due to the nature of the small and highly‐enriched DNA fragments representing DNA footprints (Figure 3a) [122, 123, 124, 125, 126]. Despite the advantages and further improvement of the ChIP‐Exo methodology and advances in bioinformatic analysis, ChIP‐Exo remains a niche technique with only ∼200 reported studies (50% from bacteria) since 2011 [127]. In summary, ChIP‐Exo represents a powerful revision of the classical nuclease protection assay coupled with high‐throughput sequencing that allows the genome‐wide characterisation of protein binding sites on DNA at near base‐pair resolution.

### 1.4 Current Challenges in the Analysis of DNA Licensing

Technological developments in recent years have resulted in fundamental biological insights. In particular, high‐throughput sequencing and optimised ChIP approaches have revealed how protein‐DNA complex assembly occurs at replication origins and what origins are associated with active DNA synthesis. However, replication origins are frequently licensed and activated in a stochastic fashion, especially in higher eukaryotes [96, 128]. Thus, population‐based methods are suboptimal for the detection of infrequent events. Single‐cell analysis is the ultimate frontier of ChIP analysis. So far, single‐cell CUT&TAG has been established for histone marks, transcription factors, and other DNA‐binding proteins [129, 130]. This has generated insights into single‐cell heterogeneity for more abundant or highly site‐specific factors, such as transcription factor OLIG2 and the cohesin complex component RAD21 [129, 130]. It will be interesting to see if this technique or another can achieve single‐cell genomic mapping of DNA replication factors. Ultimately, these data have the potential to reveal how replication origins are distributed along the genome, especially in the context of higher eukaryotes, where replication origins are less defined. Thus, single‐cell ChIP analysis would be particularly useful in the context of higher eukaryotes, where replication origins remain poorly defined [128].

It has been observed that ORC in the G2‐phase only occupies a subset of origins [82, 104]. One possibility is that insufficient ORC molecules exist in the cell. However, yeast contains a significant excess of ORC over the number of replication origins [131]. Thus, the data suggest that there are factors that restrict ORC from associating with all origins at the same time. In principle, chromatin could represent a barrier and regulator for ORC binding. However, ORC can co‐opt various chromatin remodelers to free up a nucleosome‐free region, thus suggesting that other factors are at play that restrict ORC binding in G2‐phase [132]. Interestingly, the transcription factor Fkh1 was found to recruit ORC to origins in G1‐phase, but this happens only at very few origins [133]. Hence, the reason why ORC only occupies a subset of origins in the G2‐phase remains a mystery. Addressing this question in more detail is important, as the correct spacing of replication origins is essential for genome stability.

Up to now, it was thought that budding yeast ORC only binds to sites enriched for the A‐ and B1‐elements, which occurs at replication origins and the mating type locus [134]. Recently, it was found that ORC also binds to non‐origin sequences [82]. However, binding did not occur in the G2‐phase; it was specifically observed in the G1‐phase when ORC was released from replication origins [82]. These non‐origin sequences are AT‐rich but do not share many features with A‐elements, nor is MCM2‐7 loaded to these sites effectively [82]. They occur intergenically near promoters of metabolically active genes, suggesting that they reflect open chromatin that gets transcribed in the G1‐phase [82]. Yet, the functional relevance of these non‐origin ORC binding sites is currently unknown. ORC's interaction with these sites could reflect a non‐specific DNA interaction, function as a sink, or have an additional role, such as ORC's interaction with sirtuins to regulate epigenetic silencing at the mating type locus, which remains to be discovered [135, 136]

Interestingly, ORC can bind weakly to a number of sites in the G2‐phase [82, 104]. Some of these sites could represent a novel class of initiation zones, as long‐range nanopore‐based sequencing analyses have shown that 9–20% of replication initiation events can happen outside of canonical origins [117, 137, 138]. These potentially broadly distributed and low‐efficiency origins may be used too infrequently to be detected through population‐based techniques [137, 138]. Further research is required to clarify if and how ORC recognises these low initiation zones to load MCM2‐7 complexes outside its canonical DNA binding elements.

Many insights have been gained into the DNA licensing mechanism at replication origins. Yet, the highly complex helicase activation step has not been studied using advanced techniques. Therefore, it is unclear where initial DNA unwinding occurs; is it at the B2‐ element (also known as DNA unwinding element), as initially proposed, or somewhere else [91]? Does replisome assembly lead to chromatin reorganisation, similar to DNA licensing, and what are the chromatin remodelers involved? Is it possible to detect fully assembled replisomes at replication origins? Also, the mechanisms that select the origin for activation are only partially understood. Addressing these questions represents the next frontier for the replication field.

## 2 Conclusions

The recent technological developments in the area of genomics and structural biology have propelled DNA replication research and uncovered a wealth of biological information and principles. Genomics has now achieved sufficient resolution to study protein‐DNA complex assembly at a molecular level in a genome‐wide manner. This breakthrough allows us to integrate structural and genomic‐level information to obtain fundamental insights into molecular processes. Approaches such as ChEC‐Seq, ChIP‐Exo, or CUT&TAG can be tailored to decipher novel DNA sequence motifs or the temporal order of events in reaction cascades. Indeed, the application of these approaches has revealed fundamental insights into the replicative helicase loading process. Integrating the genomics data with structural DNA shape predictions can uncover how proteins read out DNA structure to achieve specificity. In the future, AlphaFold can add another layer of structural information by generating structural models of protein‐DNA complexes [139]. As such, the integration of data from different systems across various scales can generate unified models of biological processes. In a recent example, we provided fundamental insights into the mechanisms of origin licensing and helicase loading, advancing our understanding of how DNA replication is initiated and regulated across the budding yeast genome. Our model of distributive licensing with overlapping binding sites for ORC and MCM2‐7, combined with the concept of compact origins, offers a potentially universal mechanistic explanation for genome‐wide origin licensing in eukaryotes.

## Author Contributions

Christian Speck and Luitpold Maximilian Reuter contributed to the writing of the manuscript.

## Conflicts of Interest

The authors declare no conflicts of interest.

## Data Availability

The data that support the findings of this study are openly available for MNase‐Seq [119] in the NCBI Sequence Read Archive under the accession number PRJNA663099 at https://www.ncbi.nlm.nih.gov/bioproject/663099, for ChIP‐Seq [104] in the NCBI Sequence Read Archive under the accession number SRP041314 at https://www.ncbi.nlm.nih.gov/sra/?term=SRP041314, for ChEC‐Seq [103] in the NCBI Gene Expression Omnibus data repository under the accession number GSE150800 at https://www.ncbi.nlm.nih.gov/geo/query/acc.cgi?acc=GSE150800, and for ChIP‐Exo [82] in the NCBI Gene Expression Omnibus data repository under the accession number GSE240779 at https://www.ncbi.nlm.nih.gov/geo/query/acc.cgi?acc=GSE240779.
